# The Impact of COVID-19 on Surgical Training: the Past, the Present and the Future

**DOI:** 10.1007/s12262-021-02964-2

**Published:** 2021-06-12

**Authors:** Marina Yiasemidou

**Affiliations:** 1 NIHR Academic Clinical Lecturer in General Surgery, Hull York Medical School, Hull, UK; 2 ST7 Colorectal Surgery, Bradford Teaching Hospitals, Bradford, UK

**Keywords:** COVID-19, Training, Simulation, Virtual reality

## Abstract

The COVID-19 pandemic and infection control measures had an unavoidable impact on surgical services. During the first wave of the pandemic, elective surgery, endoscopy, and ‘face-to-face’ clinics were discontinued after recommendations from professional bodies. In addition, training courses, examinations, conferences, and training rotations were postponed or cancelled. Inadvertently, infection control and prevention measures, both within and outside hospitals, have caused a significant negative impact on training. At the same time, they have given space to new technologies, like telemedicine and platforms for webinars, to blossom. While the recovery phase is well underway in some parts of the world, most surgical services are not operating at full capacity. Unfortunately, some countries are still battling a second or third wave of the pandemic with severely negative consequences on surgical services. Several studies have looked into the impact of COVID-19 on surgical training. Here, an objective overview of studies from different parts of the world is presented. Also, evidence-based solutions are suggested for future surgical training interventions.

Within 6 months of the declaration of a coronavirus SARS-CoV-2 pandemic, nearly six million cases have been confirmed worldwide and around 400,000 deaths were recorded [1]. Countries responded with unprecedented infection control and prevention measures, in an effort to contain the spread of the virus [2]. These included restrictions in unnecessary movement, widespread use of protective equipment, and sealing off national or regional borders [3].

The pandemic and infection control measures had an unavoidable impact on surgical services [4]. During the first wave of the pandemic, elective surgery, endoscopy, and ‘face-to-face’ clinics were discontinued after recommendations from professional bodies [5–10]. In addition, training courses [11, 12], examinations, conferences, and training rotations were postponed or cancelled [13]. Due to the reports of worryingly high postoperative complications, during the early days of the pandemic, several emergency pathologies were treated conservatively (e.g. appendicitis and diverticulitis) [14–17]. A further reduction of training opportunities was caused by the attempts to minimise staff members in operating theatres [18]. Many hospitals applied a ‘consultant-only’ operating policy, in order to reduce operating times and hence staff exposure [18].

Inadvertently, infection control and prevention measures, both within and outside hospitals, have caused a significant negative impact on training [19]. At the same time, they have given space to new technologies, like telemedicine and platforms for webinars, to blossom [19, 20]. Several studies have looked into the impact of COVID-19 on surgical training [19, 21–29]. Here, the author will attempt a comprehensive summary of their findings.

## An Overview

A great number of studies have reported a detrimental effect on surgical training. The majority of them described it ‘severe’ or ‘significant’ and some even ‘catastrophic’ [19, 21, 22, 28, 30–43]. These reports come both from developed [13, 21, 22, 24, 26, 31–34, 44–52] and developing countries [28, 30, 35, 53–55] with very different healthcare systems. Moreover, the results were uniform between a variety of surgical specialties; general surgery [13, 19, 21, 30, 32, 34, 37, 41, 42, 44, 47, 56, 57], orthopaedics [19, 24, 58], ENT (ear, nose, throat) surgery [19, 31, 50], vascular surgery [19, 22, 26], urology [22, 26, 53], oral and maxillofacial surgery [19, 33], and neurosurgery [19, 54, 59].

Most of the evidence provided are extracted from surveys and editorials [19, 21–29]. While most surveys have a decent response rate and a great number of responders [19, 21–29], surveys are considered to provide low-level evidence [60]. There are several reasons for this. Establishing population size estimates are important for assessing the generizability of the results of any research attempt [60]. However, there is no ideal method for population size estimation. Different methods are subject to different biases; even employing multiple approaches in an attempt to minimise these resulted in a wide variance of estimates [61]. As such, several assumptions have to be made in order to establish the ideal response rate, inevitably introducing biases [62].

In surveys, the principle of random recruitment may be violated, if authors select responders with specific characteristics from their networks [62]. Surveys are also liable to recall bias and sampling bias [62]. For instance, in the current scenario of assessing the impact of COVID-19 on surgical training, responders may misremember the number of operations performed. Moreover, in order to assess the impact of COVID-19, responders may have compared their current situation to 1 year ago, which enhances the probability of recall bias.

A degree of sampling data is unavoidable. For example, surgeons that have no access to an internet network lack access to surveys that are distributed electronically [19, 55]. Specifically, for international surveys that are distributed in the English language, sampling bias is introduced as the non-English speakers will be unable to complete the survey.

Despite the potential introduction of bias, the ‘unanimous’ results from all the surveys strengthen the findings [19, 21–29]. Also, in agreement is the demonstrated reduction of performed cases on the electronic logbook of trainees [63], which provides an ‘independent’ confirmation of the effect demonstrated in the surveys.

## Areas of Training Affected

### Theatre Exposure

Operating theatre exposure was significantly reduced [13, 19, 30, 31, 44, 53], with some authors reporting a reduction of case numbers as great as 50% [41]. Several factors contributed to this. Elective surgery came to a standstill during the first wave of the pandemic [64–66]. Emergency admissions and emergency surgery were also reduced [67]. The reduction of theatre staff numbers and ‘consultant-only operating’ policies have further reduced trainee exposure to surgery [18].

Laparoscopic surgery was identified as an aerosol-generating procedure and was hence considered high risk [68, 69]. This caused anxiety amongst theatre staff and surgeons and prompted guidelines for avoidance of laparoscopic surgery [68, 69]. While there were some exceptions [70, 71], a large number of hospitals discontinued or refrained from minimally invasive surgery [69]. As such, trainees did not get the opportunity to enhance their minimal invasive surgical skills.

Immediately after the first wave of the pandemic, the surgical community engaged in a recovery phase for surgery [72–74]. This entailed the formation of COVID-19-free pathways (‘green’ pathways) for elective patients and vigorous testing of patients and staff [74]. These processes, although necessary, are time consuming and often lead to surgical departments operating at less than 100% capacity [75]. Even to this day, delays are noticed in patient care due to testing and difficulty in self-isolating prior to elective surgery [75]. All these contribute to reduced theatre cases and hence less training opportunities for surgical learners.

The rapid development of vaccines provided a slither of hope [72] for an accelerated pathway to theatre; however, there are large discrepancies in their distribution globally [76]. Also, there are concerns that they do not prevent asymptomatic transmission [77]. Therefore, vaccination can only be used as an adjunct to self-isolation rather than a replacement.

In addition to delays outside the theatre complex, several authors report reduced workflow within it as well [78]. Several strategies were employed to increase theatre workflow during the pandemic and recovery phase [78]. Whether these are effective remains to be seen [78].

Undoubtedly, the extra steps to get the patients to theatre reduced workflow and the establishment of COVID-19-free surgical pathways [74] and have reduced the number of cases performed on an operating list. As a result, training opportunities are reduced, not only during the pandemic but the recovery phase as well.

### Outpatient Clinics

During the COVID-19 pandemic, outpatient clinics were either cancelled or converted from ‘face-to-face’ to virtual consultations [21, 22, 30, 34, 46, 55, 58, 66, 79–82]. This, initially, decreased the exposure of trainees to supervised outpatient clinics, further limiting their training opportunities [19]. On the other hand, once the initial transition period had passed, many found several advantages in virtual clinics [58, 81], accrediting them for the continuation of outpatient clinic exposure for trainees [58]. Some authors suggest that with some modifications in existing curricula, alternative interventions such as virtual clinics can form part of the routine training experience [83].

The notion of virtual outpatient clinics is at least 10 years old [84]. Their implementation saw a reduction in patients seen in ‘face-to-face’ clinic [85], improving patient experience [86]. Patient satisfaction was also high, reported in some cases as 97% [87]. A recent systematic review and meta-analysis by Chaudhry et al. [86] included 12 studies, 8 of which were randomised controlled trials comparing surgeon and patient satisfaction with ‘face-to-face’ and virtual clinics. They found no statistical difference in surgeon satisfaction (pooled OR 0.38 [95% CI 0.07 to 2.19]; p = 0.28) or in patient-reported clinical outcomes [86]. Patients reported time savings, both in respect to travel time (17 min shorter [95% CI 2 to 32]; p = 0.03) and other waiting times (180 min shorter [95% CI 78 to 281]; p < 0.001) [86]. Moreover, a randomised controlled trial by Llorens et al. [88] comparing an in-person and telemedicine clinic demonstrated significant cost savings with the virtual intervention.

Besides the expert surgeons who report at least equal satisfaction with virtual as with in-person clinics, trainees would like to see telemedicine and virtual clinics remaining as part of their practice even after the pandemic [19]. Phone or other virtual consultations can be as easily supervised as ‘face-to-face’ consultations, therefore not impeding the potential of a learning experience.

### Conferences, Training Courses, and Teaching Sessions

As a result of infection control measures aiming to reduce large gatherings, conferences, training sessions, and teaching were postponed or took place on a virtual platform [19, 56]. In a global survey conducted by our team, the trainees express a relative dissatisfaction with the virtual platforms [19]. They cited technical challenges (lack of hardware and access to a high-quality network), lack of engagement and/or interaction and inappropriate timing, as the reasons for their dissatisfaction [19]. Despite that, in the same survey, trainees did recognise the potential of these educational processes and state that they would like to see virtual conferences and courses remain as part of training after the pandemic [19]. The survey was conducted from the 23 April to the 15 May 2020, which was rather early on in the pandemic. At that time, the surgical world had to quickly adjust to the new teaching methods and perhaps was not as adequately prepared to provide high-quality virtual resources.

Surveys conducted subsequently showed extremely good trainee satisfaction with virtual conferences and teaching sessions [89–95]. Specifically, Ottesen et al. [89] reported that the virtual platform exceeded expectations of 85.7% of attendees and 100% would participate in future virtual events. There were also reports of virtual events believed to be superior to traditional conferences [90, 93].

One notable exception is the 2021 paper by Woodruff et al. [96]. While they accept that the results of phase 2/3 clinical trials are adequately reported in virtual conferences, they report fewer overall submissions [96]. They are particularly concerned that this may lead to fewer presentations of observational and post hoc analyses of clinical trials, often presented by residents, fellows, and trainees [96]. Conference presentations are essential for career progression and form an integral part of job applications [96]. The authors of this study are worried that virtual conferences hinder presentation and public speaking skills for trainees [96]. They also point out the missed opportunity for ad hoc spontaneous networking which often result in collaboration and mentorship [96]. These are all valid points which need to be addressed. The authors see hybrid conferences as a potential solution for the future [96].

### Endoscopy

Endoscopy sessions were also discontinued during the first wave of the pandemic [19, 23, 25]. This was due to concerns about viral contamination between both patients and providers of endoscopy [97]. Studies have shown that there is indeed a substantial risk of exposure and infection with respiratory diseases that can be spread via an airborne route [98]. Endoscopists are often exposed to infectious biologic samples during procedures [99]. This is particularly true due to the short physical distance between patient and endoscopist during procedures. This distance is shorter than 6 feet; the distance that SARS droplets from infectious patients can reach [100].

While endoscopy sessions were reinstated during the recovery phase of the pandemic, the numbers of procedures are reduced, again causing a negative impact on training [25, 27, 101]. Pawlak et al. [27] conducted an international survey assessing the impact of COVID-19 on endoscopy training. 93.8% out of 770 respondents reported a reduction in endoscopy case volume, with a median percentage reduction of 99% (interquartile range, 85–100%) [27]. The reduction was greatest for colonoscopy procedures [27]. The restrictions concerned not only case volume but also trainee activity (i.e. procedures were performed by experts only) [27]. A survey conducted amongst UK trainees showed similar paucity in endoscopy training [101]. The reasons cited for this were changes to institutional policy that excluded trainees from procedures (75.8%), low case volume (56.8%), and redeployment to another clinical area (47.7%) [101].

## Recovery of Training

Our group have worked on a framework for training recovery based on the results of a global survey that we conducted [19]. It emerged that trainees had concerns about the lack of guidance from training stakeholders and would like to see their mentors and trainers prioritising training at every opportunity possible [19]. Based on this and the opinion of experts, we proposed a four-stage recovery plan. This consists of:

- Guidance from national/international training stakeholders.
- Involvement of trainees, trainers, regional training programme representatives, the hospital managerial team, and the digital support team in order to discuss local implementation of guidelines and necessary adjustments that may be required locally.
- Formation of implementation team who will carry out the plan set up by above teams.
- Auditing and adjusting the plan by engaging in a ‘trial and error’ process [19].

### Alternative Methods of Training

The severe reduction of case volume is apparent in studies evaluating the impact of COVID-19 on surgical training [4, 19, 29, 39, 63, 82, 83, 101]. Therefore, methods outside the operating theatre must be sought as an adjunct to conventional training, to enhance surgical skills. Surgical simulation has been utilised for years in the surgical and other fields and was shown to be effective in enhancing surgical skills, particularly for novices [102–106]. In addition to simulation, methods like mental practice and ‘warm up’ before surgery may enhance the learning experience in the operating theatre [107–110]. Concerns about poor fidelity have now degree been resolved due to modern additive technologies such as 3D reconstruction from CT or MR images or 3D printing [107–110]. Moreover, new embalming methods made cadaveric simulation more accessible, by reducing the storage requirements and making cadavers ‘reusable’ [105, 111].

Immersive technologies are also useful for training during the pandemic [112]. These refer to virtual reality (VR), augmented reality (AR), and mixed reality (MR) [112]. Perhaps their biggest advantage is that they provide Omni-Learning; the ability to learn anywhere, anytime, with anyone [113]. AR uses holograms projected into the real-world environment [112]. This could include three-dimensional (3D) object transmission which can be viewed by a remote headset user [114]. The author in no way is suggesting that these can replace operating theatre experience, but can exponentially increase the didactic impact of every theatre session. Technologies such as these can allow for real-time streaming of operations during which the trainee can have the same optic output as their trainer (see what they see) [112], something which is of great importance in identifying efficiently and promptly the appropriate planes of dissection. Knowledge that can be put in good use the next time they are in theatre.

## Conclusion

There is little doubt that COVID-19 has significantly decreased training opportunities for surgeons [4, 19, 29, 39, 63, 82, 83, 101]. This was partially counteracted by the introduction of alternative teaching methods such as virtual teaching platforms [19, 43, 95, 96]. However, there is a long way to go to ensure that surgical training is not heavily impacted long term. This effort needs to be coordinated by training authorities nationally, with the involvement of trainees in decision-making. Alternative teaching methods should be used, not to replace, but to enhance the scarce training opportunities in existence.
